# Epidemiological Landscape of Breast Cancer in Northern India: A Narrative Review of Jammu and Kashmir

**DOI:** 10.7759/cureus.101945

**Published:** 2026-01-20

**Authors:** Gita Devi, Mittal Rathod, Mehul Kaliya, Aneri Rathod

**Affiliations:** 1 Radiodiagnosis, All India Institute Of Medical Science, Jammu, IND; 2 Community Medicine, Pandit Deendayal Upadhyay Government Medical College, Rajkot, IND; 3 Medicine, All India Institute Of Medical Science, Rajkot, IND; 4 Surgical Oncology, Gujarat Cancer Research Institute, Ahmedabad, IND

**Keywords:** breast cancer, epidemiology, jammu and kashmir, mobile mammography, molecular subtype, psychological morbidity, screening

## Abstract

Breast cancer is increasingly affecting younger women across India, with the Union Territory (UT) of Jammu and Kashmir (J&K) experiencing a similar shift. Limited regional data and poor screening access contribute to late diagnosis and high psychosocial burden, necessitating a synthesized evaluation of existing evidence. The objective of this review is to summarize and critically appraise the published literature on breast cancer in Jammu and Kashmir, with a specific focus on its epidemiology, clinical characteristics, molecular subtype distribution, awareness levels, and psychosocial determinants. A narrative review approach was adopted. Studies published in English and conducted within J&K were searched through PubMed, MEDLINE, and Google Scholar. Eligible studies included those reporting epidemiology, staging, molecular patterns, risk awareness, and mental-health outcomes. Data were extracted and synthesized according to the study characteristics. A total of 12 studies were included. Findings revealed a younger pattern of onset, with most cases detected between the late 30s and early 60s. Late-stage presentation (≥ stage III) was predominant, especially in Kashmir. Infiltrating ductal carcinoma and HR+/HER2- subtype were the most common. Awareness and screening rates were low, particularly among women >40 years. High levels of depression, anxiety, and post-traumatic stress disorder (PTSD) were reported among patients in Jammu. Mobile mammography initiatives showed high feasibility but poor follow-up linkage. Breast cancer in J&K is characterized by late diagnosis, distinct molecular patterns, limited awareness, and high psychological burden. Strengthening community-level screening, establishing population-based cancer registries, expanding mobile mammography programs, and integrating psycho-oncology into cancer care are crucial for improving early detection and outcomes in the UT.

## Introduction and background

Breast cancer is the most common malignancy among women worldwide, and its burden is rising rapidly across India, including the Union Territory (UT) of Jammu and Kashmir (J&K) [1-3]. The incidence of breast cancer in India has increased by nearly 39% between 1990 and 2016, reflecting a significant epidemiological transition [4]. In 2020, breast cancer accounted for 13.5% of all cancer cases and 10.6% of all cancer-related deaths in the country [2]. Recent trends also indicate a shift toward a younger age at diagnosis, with a growing proportion of cases presenting in pre-menopausal women. Detecting cases at advanced stages leads to higher treatment costs and worse clinical outcomes, underscoring the need for early identification.

The Government of India, through the National Programme for Prevention and Control of Cancer, Diabetes, Cardiovascular Diseases, and Stroke (NPCDCS), has outlined operational guidelines and standardized recommendations for breast cancer screening and early detection [5, 6]. Despite these initiatives, substantial challenges persist. Delays in diagnosis, inadequate referral pathways, limited community awareness of risk factors and screening methods, and suboptimal knowledge even among healthcare professionals continue to impede early detection [1, 6]. Socio-cultural barriers, stigma, and unequal access to diagnostic facilities further exacerbate these gaps.

A preliminary scan of available literature reveals a marked imbalance between the Jammu and Kashmir divisions. Research from Jammu is modest, focusing primarily on psychiatric comorbidities, awareness, and general cancer patterns [7-9]. In contrast, Kashmir has produced more extensive biomedical literature, including phenomenological accounts, analyses of treatment failures, epidemiological assessments, and clinicopathological profiles [10-14]. This asymmetry highlights critical evidence gaps, particularly regarding epidemiological determinants, demographic distribution, clinical characteristics, and screening practices in Jammu.

Jammu and Kashmir was selected for focused review due to its unique geopolitical and health-system context. Evidence from fragile and conflict-affected settings indicates that prolonged instability adversely affects continuity of care, preventive services, and access to early diagnosis [15]. Geographic barriers and sociocultural determinants further contribute to delayed breast cancer detection in India [16]. Recent National Council on Radiation Protection and Measurements (NCRP)-based analyses highlight persistent regional gaps in cancer surveillance and burden estimation, underscoring the need for region-specific synthesis [17].

Given these considerations, this review synthesizes available evidence on breast cancer in J&K, examining epidemiology, risk factors, screening and diagnostic pathways, and management. A clearer understanding of the regional burden can help strengthen early detection, improve outcomes, and guide policy and public health interventions tailored to the unique context of the UT.

The absence of a formal risk of bias assessment represents a limitation of this narrative review and should be considered when interpreting the findings.

## Review

Methods

This review adopted a narrative synthesis approach to summarize current evidence on breast cancer epidemiology, risk factors, screening practices, and diagnostic pathways in the Union Territory of Jammu and Kashmir. A narrative review methodology was adopted due to the limited number of region-specific studies from the Union Territory of Jammu and Kashmir and marked heterogeneity in study designs and outcome reporting. As the available evidence included diverse methodologies - ranging from hospital-based and registry studies to qualitative and community surveys - a systematic review or PRISMA-based meta-analysis was not feasible. A narrative approach was therefore used to synthesize fragmented evidence and provide contextual clinical and public health interpretation relevant to this region. A comprehensive literature search was performed on PubMed, MEDLINE, and Google Scholar for studies published between January 2000 and December 2024, using a combination of Medical Subject Headings (MeSH) and free-text keywords such as "breast cancer", "carcinoma breast", "Jammu", "Kashmir", "epidemiology", "risk factors", "screening", and "clinicopathological profile", with Boolean operators (AND, OR). Additionally, reference lists of included studies were manually screened to identify relevant articles, while grey literature, non-indexed publications, and dissertations were excluded to maintain scientific rigor. Two independent reviewers screened titles and abstracts, followed by full-text assessment of potentially eligible articles. Duplicate records were removed manually. Disagreements were resolved by discussion and consensus.

Studies were included if they focused specifically on breast cancer in the Jammu and Kashmir region, reported epidemiological trends, risk factors, clinicopathological profiles, or screening and diagnostic practices, were original research articles published in peer-reviewed journals, and were available in English. Studies were excluded if they did not pertain to Jammu and Kashmir, addressed cancers other than breast cancer, were review articles, commentaries, editorials, or conference abstracts, or were non-English publications or lacked adequate scientific rigor.

Data extraction was carried out using a predefined template that captured author details, year of publication, study design and journal, sample size and study setting, epidemiological indicators such as incidence, prevalence, mortality, and stage at diagnosis, assessed risk factors, and information on screening and diagnostic practices. Two reviewers independently extracted data to ensure accuracy and minimize bias. Owing to methodological heterogeneity across studies, a narrative synthesis approach was employed, with findings organized thematically to highlight epidemiological patterns, risk determinants, screening practices, and regional disparities between Jammu and Kashmir. Quality assessment focused on journal indexation, clarity of methodology, appropriateness of sampling, and completeness of reporting, though no formal risk-of-bias tool was applied because of the diversity in study designs. Ethical approval was not required as the review analyzed secondary data from published sources, and all studies were duly cited. The review was not registered on PROSPERO, as narrative reviews are generally not eligible for registration.

**Figure 1:**
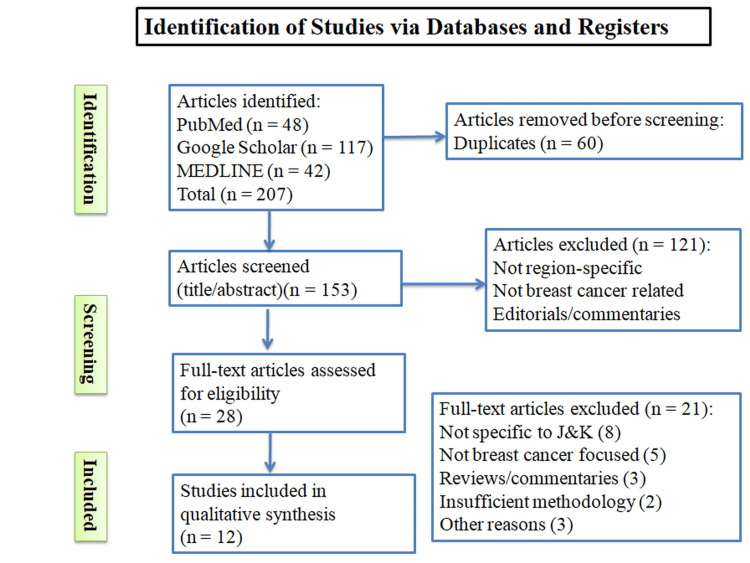
PRISMA flow diagram showing identification, screening, eligibility assessment and inclusion of studies PRISMA - Preferred Reporting Items for Systematic Reviews and Meta-Analyses

A total of 207 records were identified through database searches (PubMed: 48, Google Scholar: 117, and MEDLINE: 42), and an additional six records were found from other sources. After removing duplicates, 153 records were retained for screening, all of which underwent title and abstract review. Of these, 121 were excluded, and 32 full-text articles were sought for retrieval. Four full texts could not be obtained, leaving 28 articles assessed for eligibility. Twenty-one articles were excluded, mainly for not being specific to Jammu and Kashmir (n=8), not focusing on breast cancer (n=5), being reviews or commentaries (n=3), having insufficient methodology (n=2), or other reasons (n=3). Ultimately, 12 studies met the inclusion criteria and were incorporated into the qualitative synthesis (Figure 1).

Results

Epidemiological Characteristics and Age Profile

The literature indicates a distinct epidemiological pattern of breast cancer in the Union Territory of Jammu and Kashmir, characterized by a younger age of onset than commonly documented in high-income regions. Evidence from Kashmir shows that most women present with the disease in their late 30s to early 60s, suggesting a predominantly pre-menopausal burden [12]. The age distribution reported in clinical cohorts from tertiary settings aligns with national trends of rising breast cancer among younger Indian women. Registry-based findings from Jammu and Kashmir further establish breast cancer as a major malignancy among females; however, granular clinical age data remain limited within registry-only outputs [14]. Table 1 provides an overview of studies documenting such characteristics, while Table 2 emphasizes early menarche as a recurrent epidemiological determinant in Kashmir cohorts, reflecting shifting reproductive profiles within the region. As summarized in Table 3, this evidence demonstrates greater clinical depth from Kashmir, whereas research output from Jammu remains focused on psychosocial aspects rather than epidemiological stratification.

**Table 1:** Characteristics of included studies (Jammu and Kashmir, 2017-2024) UT - Union Territory; J&K - Jammu and Kashmir; HADS - Hospital Anxiety and Depression Scale; PSS-10 - Perceived Stress Scale; IHC -immunohistochemistry; BI-RADS - Breast Imaging Reporting and Data System

Sr. No.	Year	Journal	Title and authors	Study type (n)	Study population	Study area	Study tool	Objectives
1	2023	JK Science	Kumari et al. Prevalence and determinants of anxiety disorders among females with breast cancer attending a tertiary care hospital in Jammu [7]	Hospital-based cross-sectional (n=275)	Females ≥ 18 years with confirmed breast cancer	Jammu (Tertiary Care Hospital)	Structured questionnaire (anxiety screening)	To determine the prevalence of anxiety disorders and assess socio-demographic and clinical determinants.
2	2023	J Cancer Res Ther	Mir et al. Psychiatric comorbidities and breast cancer: a study from Jammu region of UT of J&K, India [8]	Cross-sectional (n=205)	Females ≥ 18 years with breast cancer	Jammu (Regional Cancer Centre)	Self-designed proforma + HADS + PSS-10	To assess psychiatric comorbidities and associations with demographic/clinical factors.
3	2020	Int Health Res J	Javaid et al. Knowledge and practice of breast cancer screening and awareness of its risk factors among reproductive women of Jammu & Kashmir [9]	Community-based cross-sectional (n=387)	Women aged 15-45 years from he general population	Jammu and Kashmir (urban + rural)	Structured questionnaire (awareness and screening)	To assess awareness of risk factors and screening practices among reproductive-age women.
4	2021	Health Promot Int	Hamid et al. Lived experiences of women suffering from breast cancer in Kashmir: a phenomenological study [10]	Qualitative phenomenological (n=12)	Women ≥ 18 years; diagnosed ≥ 6 months earlier	Kashmir (various districts)	In-depth interviews (snowball + theoretical sampling)	To explore lived experiences, challenges, and coping strategies of women with breast cancer.
5	2019	Ann Int Med Den Res	Paul et al. Patterns of failure in breast cancer patients of Kashmir Valley [11]	Retrospective observational (n=212)	Biopsy-proven breast cancer patients treated at tertiary centre	Kashmir Valley	Hospital records + follow-up data	To analyze local, regional, and distant failure patterns after treatment.
6	2021	J Community Med	Khursheed et al. Epidemiological studies on breast cancer in Kashmir Valley [12]	Secondary data analysis (n=226)	Female patients aged 19-78 years, pathological breast cancer	Kashmir Valley	Hospital cancer registry dataset	To describe epidemiological characteristics of breast cancer cases attending tertiary centre.
7	2017	Surg Sci	Mir et al. Clinicopathological profile of breast cancer patients at a tertiary care hospital in Kashmir Valley [13]	Hospital-based cross-sectional observational (total malignant cases n=34)	Males and females, aged 22-60 years, histopathologically confirmed breast cancer	Kashmir Valley (GMC Srinagar)	Clinical evaluation, histopathology, record review	To determine demographic, clinical, pathological, and staging profile of breast cancer in Kashmir.
8	2017	Indian J Comput Sci Eng	Nisa et al. Survey of patients with cancer in Jammu & Kashmir: based on hospital registry records [14]	Hospital-based registry retrospective survey (total n=6359; breast cancer subset n=234)	All cancer patients; breast cancer subset analyzed	Jammu and Kashmir (four major hospitals)	Review of hospital registry records	To describe cancer site distribution, age/sex distribution among cancers, including breast cancer in J&K.
9	2024	J Cancer Res Ther	Banday et al. Receptor subtype and outcomes of breast cancer - single-center experience from North India [18]	Retrospective cohort (n=944)	Female, biopsy-proven breast cancer patients treated 2014-2018	Kashmir (SKIMS, Srinagar)	Hospital records; pathology and IHC data; follow-up records	To determine molecular subtype distribution and compare survival across subtypes.
10	2024	BMC Public Health	Gupta et al. The Chiraiya Project: retrospective analysis of breast cancer detection gaps via mobile mammography in Jammu Province, India [19]	Retrospective observational screening (n=1505 screened)	Women ≥ 40 years, general population	Jammu Province, J&K (urban + rural)	Mobile mammography; BI-RADS + structured database	To analyze detection gaps, follow-up adherence, urban-rural uptake, feasibility of mobile mammography in underserved areas.

**Table 2:** Synthesis of major findings from breast cancer studies in Jammu and Kashmir (2017-2024) J&K - Jammu and Kashmir; PTSD - post-traumatic stress disorder; TNBC - triple-negative breast cancer

Study	Domain of Study	Key Findings	Implications for J&K
Kumari et al. [7]	Psychological morbidity (anxiety disorders)	High prevalence of panic disorder, PTSD, and agoraphobia; rural residence and age/family type associated with higher PTSD/agoraphobia rates.	Need for integrated mental-health services for oncology patients.
Mir et al. [8]	Psychiatric comorbidity (depression, anxiety, stress)	High burden of depression and anxiety; associated with parity, hospital visits, duration since diagnosis, treatment type.	Psychological screening should be integrated into routine breast cancer care.
Javaid et al. [9]	Awareness and screening practices (general population)	Low awareness of symptoms and risk factors; poor screening uptake; younger women are somewhat more informed.	Targeted awareness campaigns and community education, especially for older women needed.
Hamid et al. [10]	Lived experiences and coping mechanisms	Women reported serious psychological, social, financial, and emotional challenges; coping through spirituality, family support, and emotional venting.	Psychosocial support and stigma-reduction interventions essential.
Paul et al. [11]	Patterns of failure (post-treatment)	Significant rates of local, regional, and distant recurrences; infiltrating ductal carcinoma is common.	Strengthened follow-up protocols and early detection of recurrence required.
Khursheed et al. [12]	Epidemiological profile	Younger age at diagnosis; early menarche identified as risk factor; majority present at advanced stages.	Urgent need for screening, awareness, and early-detection strategies.
Mir et al. [13]	Clinicopathological profile	High proportion presenting in stage III; infiltrating ductal carcinoma predominant.	Indicates delayed presentation - emphasizes need for community outreach and timely Refral.
Nisa et al. [14]	Cancer registry-based survey	Breast cancer among top reported cancers; data limited to hospital-based registry; under-reporting probable.	Population-based cancer registry establishment needed for accurate disease burden assessment.
Banday et al. [18]	Molecular subtype distribution and outcomes	HR+/HER2- subtype most common (44.5%); TNBC 15.7%, associated with worst survival outcomes.	First molecular profile for J&K - informs subtype-specific treatment and prognosis.
Gupta et al. [19]	Screening-gap analysis via mobile mammography	Screening outreach to 1,505 women; detection gaps, poor follow-up, urban-rural disparities were observed.	Mobile screening shows promise, but follow-up and Refral systems must be strengthened.

**Table 3:** Comparative epidemiological and clinical profile of breast cancer in Jammu vs. Kashmir TNBC - triple-negative breast cancer

Parameter	Findings from Jammu-based studies [7-9, 16]	Findings from Kashmir-based studies [10-15]	Overall Interpretation/trend
Age at diagnosis	Limited data - mainly psychological studies, age ≥ 18 years; no precise staging or histopathology data	Clinical studies report diagnosis between late 30s and early 60s; younger onset cases exist	Early-onset trend seen in Kashmir; Jammu lacks clinical cohort data
Stage at presentation	Not reported	High proportion present at stage III (70-73%)	Late-stage presentation is common; region-wide challenge
Most common symptoms	Not reported	Breast lump most frequent; nipple discharge, skin changes also observed	Clinical symptom and presentation data predominantly from Kashmir
Histopathology	Not available	Infiltrating ductal carcinoma predominant in pathology reviews	Infiltrating ductal carcinoma is dominant subtype in region; clinical data from Kashmir essential
Molecular subtypes	Not reported	HR+/HER2- most common; TNBC significant (15.7%) with poorer prognosis	Molecular profiling limited to Kashmir; critical data gap for Jammu
Psychiatric and psychosocial impact	High anxiety, depression, stress [7-9]	High psychosocial burden described in phenomenological study [10]	Psychological distress common across both divisions; need for mental-health integration
Awareness and screening uptake	Low awareness; poor screening practices [9]	Mobile screening shows reach but follow-up gaps and rural-urban disparity [18]	Screening coverage limited; both community awareness and structured programmes needed
Recurrence / treatment failure patterns	Not reported	Documented local, regional, distant failures [11]	Follow-up care and surveillance critical post-treatment
Cancer registry and surveillance data	Registry-based survey includes cancer distribution but limited clinical detail	Hospital-based epidemiological and pathology data [12, 13, 18]	Kashmir offers richer clinical datasets; Jammu relies on registry - need integrated registry + clinical data
Screening initiatives and innovation	No community-level screening innovation reported	Mobile mammography implemented in underserved areas [19]	Mobile screening is a viable model for broader coverage; needs support for follow-up

Stage at Presentation and Disease Progression

Across available clinical datasets, late-stage diagnosis emerges as a dominant challenge. Studies from tertiary centers in Kashmir reveal that more than 70% of patients present with stage III breast cancer at diagnosis, whereas only around 27% report at stage II [13]. This skewed stage distribution suggests poor symptom recognition, delayed health-seeking behavior, and limited access to structured screening pathways. Complementary evidence from a treatment outcome analysis underscores high rates of local, regional, and distant failures, suggesting that delays likely extend beyond initial diagnosis to include inadequate post-treatment surveillance and follow-up [11]. These patterns highlight critical weaknesses within both early detection and survivorship care in the UT. Table 2 consolidates these failure patterns, while Table 3 reinforces that such clinical insights originate exclusively from Kashmir, leaving a significant evidence gap on disease staging and progression in Jammu.

Molecular Profile and Histopathological Characteristics

Histopathological evaluation across Kashmiri cohorts consistently identifies infiltrating ductal carcinoma (IDC) as the most common tumor subtype [13], a pattern concordant with Indian and global distributions. However, the molecular subtype distribution adds an important layer to regional clinical management. A large tertiary-center cohort study from Kashmir documented that hormone receptor-positive, HER2-negative (HR+/HER2-) tumors constituted the predominant subtype (44.5%), whereas triple-negative breast cancer (TNBC) accounted for 15.7% of all cases [18]. TNBC was associated with significantly poorer survival outcomes and more aggressive disease trajectories, indicating the need for timely subtype-specific management. These molecular findings, presented in Table 2, offer critical prognostic and therapeutic implications for the region. As seen in Table 3, such molecular datasets are currently exclusive to Kashmir, indicating the absence of comparable data from Jammu and underscoring the need for region-wide tumor profiling.

Awareness Levels, Screening Practices, and Accessibility Initiatives

Despite evidence of younger disease onset, community-level awareness and screening remain disappointingly low. A cross-sectional population-based survey reported inadequate knowledge of breast cancer symptoms, risk factors, and screening modalities among women, particularly those above 40 years of age [9]. Even though younger women demonstrated relatively higher awareness, this did not translate into sustained screening practices. These behavioral inconsistencies contribute directly to delayed presentation, as reflected in the high proportion of late-stage diagnoses [13]. Efforts to bridge diagnostic gaps have been demonstrated through the "Chiraiya" mobile mammography project in Jammu Province, which screened 1505 women aged ≥40 years and revealed disparities in rural uptake and poor follow-up adherence [19]. Table 1 captures the screening intervention methodology, while Table 3 highlights the initiative's implications for equitable screening access. This model illustrates that mobile units may offer scalable public health solutions in geographically challenging areas of the UT, provided systematic recall and referral mechanisms are strengthened.

Psychosocial and Mental Health Burden

The psychosocial dimension of breast cancer care is particularly visible in research from Jammu, where multiple studies highlight a high burden of anxiety, depression, stress, and post-traumatic stress disorders among patients [7, 8]. Determinants include rural residence, treatment modality, time since diagnosis, parity, and family structure. Qualitative exploration from Kashmir further reveals the lived emotional distress associated with breast cancer, including stigma, socioeconomic insecurity, and reliance on coping mechanisms such as spirituality and family support [10]. As summarized in Tables 1 and 2, these findings make a compelling case for integrating structured psychological screening and support into oncology services across the UT. Table 3 demonstrates that while clinical data are richer for Kashmir, psychosocial evidence is concentrated in Jammu, highlighting the need for comprehensive bilateral data integration.

Discussion

While the reviewed studies frequently highlight differences between Jammu and Kashmir, these differences are best understood in the context of broader demographic, socioeconomic, and health-system characteristics as depicted in Table 4.

**Table 4:** Contextual factors potentially influencing regional differences between Jammu and Kashmir

Domain	Jammu	Kashmir	Relevance to breast cancer research
Urbanization	Higher	Lower	Affects access to screening and diagnostics
Major cities	Jammu	Srinagar	Concentration of tertiary care
Health facilities	Relatively better distributed	Concentrated in few centers	Influences reporting and early diagnosis
Female literacy	Relatively higher	Lower in some districts	Affects awareness and care-seeking
Research institutions	More medical colleges	Fewer centers	Influences volume of publications
Cancer surveillance	Limited	Limited	Under-reporting in both regions

Jammu is relatively more urbanized, with higher population concentration around major cities such as Jammu city, and comparatively better access to tertiary health-care facilities. In contrast, Kashmir has a higher proportion of rural and geographically dispersed populations, with access to specialized oncology services concentrated in a limited number of urban centers. Differences in female literacy, health-seeking behavior, and availability of diagnostic infrastructure may further influence stage at presentation and reporting of breast cancer cases. Variations in medical education institutions, research activity, and cancer surveillance capacity across the two regions may also contribute to the observed imbalance in published literature. These contextual factors should be considered when interpreting apparent regional differences in breast cancer epidemiology [15].

Breast cancer in Jammu and Kashmir reflects an evolving epidemiological profile similar to the recent shifts observed globally, where rising incidence among younger women has been attributed to changing reproductive behaviors, hormonal influences, obesity, and delayed childbearing [20]. Regional findings in Kashmir showing early-age onset and premenopausal diagnosis align with this global risk-transition model [12-14]. Furthermore, the predominance of HR+/HER2- tumors and a substantial proportion of TNBC cases in Kashmir highlight the growing importance of biomarker-based management, which is increasingly emphasized in international breast cancer care frameworks [10].

In addition to clinical gaps, psychosocial morbidity is a prominent and under-recognized dimension in breast cancer care within the UT. High prevalence of depression, anxiety, and PTSD among patients in Jammu [7, 8] echoes international evidence asserting that quality of life and treatment response are negatively affected when emotional distress is untreated [21]. These findings reinforce the need for an integrative care model in J&K that incorporates psycho-oncology services alongside conventional therapeutics.

Delayed presentation remains a persistent challenge in J&K, with over two-thirds of women presenting in stage III [13]. This parallels low-resource settings worldwide, where stigma, limited diagnostic infrastructure, and socioeconomic factors impede timely screening and care [22]. The success of the mobile mammography initiative in Jammu demonstrates that resource-appropriate screening approaches can improve early detection when properly linked with follow-up and referral pathways [16]. Such strategies reflect global recommendations advocating community-based outreach and mobile diagnostic models as viable solutions for underserved populations [22].

Therefore, the current evidence underscores three critical public health priorities for J&K: establishing a population-based cancer registry, expanding decentralized community-level screening programs, and incorporating structured psychological support into oncologic care. Implementing these components can narrow the disparity between Kashmir and Jammu research outputs and align J&K's health response with global cancer control goals.

## Conclusions

Breast cancer in Jammu and Kashmir demonstrates a rising burden marked by younger age of onset, late-stage diagnosis, inadequate awareness, and substantial psychosocial morbidity. Kashmir contributes more evidence on clinical, pathological, and molecular profiles, whereas Jammu reflects significant psychological and community-based gaps. With most cases presenting beyond stage II, strengthened community screening, decentralized diagnostic services, and mental health integration are urgently required. Evidence from this review highlights the need to establish population-based cancer surveillance, multidisciplinary oncology services, and context-specific screening pathways, including mobile mammography programs, to improve early detection and reduce mortality. Such targeted interventions will help address the emerging epidemiological shift and bridge the disparity in breast cancer research and management across the Union Territory.

## References

[1] Gupta A, Shridhar K, Dhillon PK (2015). A review of breast cancer awareness among women in India: cancer literate or awareness deficit?. Eur J Cancer.

[2] Mathur P, Sathishkumar K, Chaturvedi M (2020). Cancer statistics, 2020: report from National Cancer Registry Programme, India. JCO Glob Oncol.

[3] Gupta A, Puri I, Gupta M (2021). Patterns of cancer in males and females in Jammu Region. JK Science.

[4] Mehrotra R, Yadav K (2022). Breast cancer in India: present scenario and the challenges ahead. World J Clin Oncol.

[5] (2021). National Programme for Prevention and Control of Cancer, Diabetes, Cardiovascular Diseases & Stroke (NPCDCS) Operational Guidelines (2013-17). Cancer, Diabetes, Cardiovascular Diseases & Stroke (NPCDCS) Operational.

[6] Mishra GA, Pimple SA, Mittra I, Badwe RA (2021). Screening for breast cancer: cost-effective solutions for low- & middle-income countries. Indian J Med Res.

[7] Kumari R, Mir MT, Mahajan R (2023). Prevalence and determinants of anxiety disorders among females with breast cancer attending a tertiary care hospital in Jammu. JK Science.

[8] Mir MT, Kumari R, Gupta RK, Sharma R, Gul N, Langer B (2023). Psychiatric comorbidities and breast cancer: a study from Jammu region of UT of J&amp;K, India. J Cancer Res Ther.

[9] Javaid M, Swaminathan J (2020). Knowledge and practice of breast cancer screening and awareness of its risk factors among reproductive women of Jammu and Kashmir. Int Health Res J.

[10] Hamid W, Jahangir MS, Khan TA (2021). Lived experiences of women suffering from breast cancer in Kashmir: a phenomenological study. Health Promot Int.

[11] Paul ZA, Banday SZ, Afroz F (2019). Patterns of failure in breast cancer patients of Kashmir Valley. Ann Int Med Den Res.

[12] Khursheed WA, Thakur N, Sheikh K (2021). Epidemiological studies on breast cancer in Kashmir Valley. J Community Med.

[13] Mir MA, Manzoor F, Singh B (2017). Clinicopathological profile of breast cancer patients at a tertiary care hospital in Kashmir Valley. Surg Sci.

[14] Nisa KU, Kumar R (2017). Survey of patients with cancer in Jammu & Kashmir based on hospital registry records. Indian J Comput Sci Eng.

[15] Malvia S, Bagadi SA, Dubey US, Saxena S (2017). Epidemiology of breast cancer in Indian women. Asia Pac J Clin Oncol.

[16] Kulothungan V, Ramamoorthy T, Sathishkumar K, Mohan R, Tomy N, Miller GJ, Mathur P (2024). Burden of female breast cancer in India: estimates of YLDs, YLLs, and DALYs at national and subnational levels based on the national cancer registry programme. Breast Cancer Res Treat.

[17] (2025). World Health Organization. Accessing essential health services in fragile, conflict-affected and vulnerable settings. https://www.who.int/activities/accessing-essential-health-services-in-fragile-conflict-affected-and-vulnerable-settings.

[18] Banday SZ, Ayub M, Rasool MT (2024). Receptor subtype and outcome of breast cancer - single-center experience from North India. J Cancer Res Ther.

[19] Gupta G, Jamwal N, Gupta R (2024). The Chiraiya project: a retrospective analysis of breast cancer detection gaps addressed via mobile mammography in Jammu Province, India. BMC Public Health.

[20] Sung H, Ferlay J, Siegel RL, Laversanne M, Soerjomataram I, Jemal A, Bray F (2021). Global cancer statistics 2020: GLOBOCAN estimates of incidence and mortality worldwide for 36 cancers in 185 countries. CA Cancer J Clin.

[21] Nicolis O, De Los Angeles D, Taramasco C (2024). A contemporary review of breast cancer risk factors and the role of artificial intelligence.. Frontiers in Oncology.

[22] Islam RM, Billah B, Hossain MN, Oldroyd J (2017). Barriers to cervical cancer and breast cancer screening uptake in low-income and middle-income countries: a systematic review. Asian Pac J Cancer Prev.

